# Measuring Social and Role Recovery: Barbara Cornblatt and Colleagues’ Global Functioning Scales and Their Role in Psychosocial Interventions for Clinical High-Risk and Early Psychosis

**DOI:** 10.1093/schbul/sbaf259

**Published:** 2026-08-11

**Authors:** Jason Schiffman, Katherine Wislocki, Emily Petti, Miranda A Bridgwater, Ceouna Hegwood, Shadi Sharif, Maksim Giljen, Alison Boos, Sarah Coscolluela, Elizabeth A Martin, Vijay A Mittal

**Affiliations:** Department of Psychology, 4201 Social & Behavioral Sciences Gateway, University of California Irvine, Irvine, CA 92697-7085, United States; Department of Psychology, 4201 Social & Behavioral Sciences Gateway, University of California Irvine, Irvine, CA 92697-7085, United States; Department of Psychology, 4201 Social & Behavioral Sciences Gateway, University of California Irvine, Irvine, CA 92697-7085, United States; Department of Psychology, 4201 Social & Behavioral Sciences Gateway, University of California Irvine, Irvine, CA 92697-7085, United States; Department of Psychology, 4201 Social & Behavioral Sciences Gateway, University of California Irvine, Irvine, CA 92697-7085, United States; Department of Psychology, 4201 Social & Behavioral Sciences Gateway, University of California Irvine, Irvine, CA 92697-7085, United States; Department of Psychology, 4201 Social & Behavioral Sciences Gateway, University of California Irvine, Irvine, CA 92697-7085, United States; Department of Psychology, 4201 Social & Behavioral Sciences Gateway, University of California Irvine, Irvine, CA 92697-7085, United States; Department of Psychology, 4201 Social & Behavioral Sciences Gateway, University of California Irvine, Irvine, CA 92697-7085, United States; Department of Psychology, 4201 Social & Behavioral Sciences Gateway, University of California Irvine, Irvine, CA 92697-7085, United States; Department of Psychology, Northwestern University, Evanston, IL 60208, United States

**Keywords:** clinical high-risk for psychosis, early psychosis, early intervention, global functioning scales, social functioning, role functioning

## Abstract

**Background:**

Functional outcomes, social and role, are key to the lives of people at clinical high-risk for psychosis (CHR) and with early psychosis. Recognizing this, Barbara Cornblatt and colleagues created the Global Functioning: Social (GF:S) and Global Functioning: Role (GF:R) scales. In doing so, they provided developmentally appropriate, domain-specific measures of functioning. Primary goals of this article were to (1) quantify and describe the scales’ usage, and (2) summarize key findings from studies employing the scales in psychosocial intervention.

**Study Design:**

We systematically reviewed studies citing Cornblatt et al., which introduced GF:S and GF:R. Data were extracted with Elicit (AI) and verified manually.

**Study Results:**

Of 742 citations from Google Scholar, 334 studies explicitly used the scales and were included for review. Of these, 39 examined psychosocial interventions across CHR, first episode psychosis (FEP), or both. Patterns of findings suggest that functional change is domain- and stage-specific. In CHR samples, interventions that explicitly targeted social functioning more often yielded improvements on the GF: Social scale than on the GF: Role scale. In FEP samples, role functioning showed clearer gains, particularly in multi-component interventions that combined psychosocial and pharmacological or exercise-based approaches.

**Conclusions:**

The GF:S and GF:R scales have shaped interventions and research. The development of these tools reflected a thoughtful vision from Dr. Cornblatt and colleagues that placed functioning at the center of early intervention, advancing a more person-centered field. This review highlights the broad reach of the GF scales, as well as Dr. Cornblatt’s legacy as a pioneer who shaped the field of early psychosis.

## Introduction

Mental health diagnoses are often primarily defined by the presence of symptoms, similar to many conditions (eg, general medicine) in which subjective experiences (eg, pain or fatigue) and other verbal or behavioral reports are the primary indicators of underlying pathology. This is certainly the case for many disorders associated with psychosis, with psychosis-spectrum disorders generally identified by the presence of hallucinations, delusions, and disorganization.^1^^,^^2^ Consistent with this tradition, historically, mental health treatment has focused on symptom reduction, with symptom severity driving diagnostic and treatment decisions. In other words, symptoms are often the primary way the mental health field conceptualizes concerns and, subsequently, what the field tends to prioritize in the provision of care. Although not without its strengths, this approach often fails to capture the experiences of impacted individuals and the impact that their mental health challenges may have on their daily lives.^3^^,^^4^

In recent years, a complementary perspective has gained momentum that shifts focus from symptom reduction to the real-world functioning of individuals navigating mental health challenges.^5^^,^^6^ Functioning refers to how effectively individuals navigate life roles (eg, work, school) and social relationships.^7^ Whereas mental health professionals often prioritize symptoms, it is not uncommon for clients to prioritize functional outcomes, such as improved quality of life, maintaining relationships, and employment, over symptom management alone.^8-10^ There is a growing recognition that an individual can experience clinical symptoms yet still lead a meaningful, satisfying, and abundant life through effective social and role functioning.^11-13^ The emphasis on functioning is in large part due to leaders in the field who recognized the need for better ways to understand and support the lives of individuals in the early stages of psychosis. Barbara Cornblatt is among these leaders whose legacy we reflect on in this article.

Identifying individuals at clinical high-risk for psychosis (CHR) is key to intervening early to potentially reduce the severity or prevent the onset of full-threshold psychosis.^14^ Early intervention is not just important for symptom attenuation, but also for maintaining functioning that can deteriorate as mental health challenges persist.^15^^–^^17^ Therefore, the rationale for considering functioning as an ongoing variable of interest in CHR is strong. Additionally, functional impairment often precedes and predicts transition to psychosis, speaking to the importance of measuring this construct as an early predictor of outcome in CHR populations.^18^

While the psychosis field has a long history of functioning assessment, traditional tools were not designed for those in the earlier phases of illness progression. Existing global scales often lacked sensitivity to developmental nuances in adolescents and young adults, the primary demographic of interest in CHR research.^7^ Furthermore, functioning measures historically combined social and role domains into a single global score (eg, GAF, SOFAS).^1^ Combining these domains, however, might miss meaningful variation. Early evaluations indicated that social and role functioning are not interchangeable: social functioning tends to remain stable and is linked to later psychosis, whereas role functioning is comparatively labile and responsive to treatment and environmental supports.^7^ Additionally, the absence of specific anchors to differentiate levels of impairment in adolescents and young adults posed significant measurement challenges for one of the most important constructs of interest in CHR research and clinicalcare.

These challenges are especially relevant given the movement toward client-driven care (eg, shared decision-making), which emphasizes functional outcomes valued by clients. These functional outcomes, which could include employment, social integration, and independence, are influenced by the enhanced knowledge, engagement, and attitudes toward treatment promoted through a fuller understanding of a client’s social and role functioning.^19^ A key element of success for effective approaches that prioritize functioning is the employment of reliable and valid measures of functioning for the population of interest—in this case, youth and young adults in the transition-aged youth/emerging adulthood developmental window who also meet criteria for psychosisrisk.

Recognizing this need for a developmentally tailored measure of functioning, Barbara Cornblatt and colleagues within the North American Prodrome Longitudinal Study (NAPLS) consortium^20^^,^^21^ spearheaded the development of specialized scales to standardize assessment.7 Referred to as the Global Functioning: Social (GF:S) and Global Functioning: Role (GF:R) scales, these sibling measures were designed for individuals at CHR and probe social and role functioning domains separately using developmentally appropriate anchors.

The GF:S and GF:R scales were introduced in 2007 as 2 clinician-rated scales.^7^ Both use a 1-10 rating format, with clear developmental anchors to assess functional impairment (1 = “Extreme dysfunction” and 10 = “Superior functioning”). Ratings are given for current functioning, lowest functioning in the past year, and highest functioning in the past year, allowing for assessment over time. Initial psychometric evaluations demonstrated robust inter-rater reliability and concurrent validity with symptom severity and overall functioning.^7^ These early and strong psychometric properties for an obviously needed tool would provide the foundation for a wealth of studies to follow, as well as their integration into treatment planning, progress monitoring, and program evaluation.^22^

Since publication, the GF scales have become widely integrated into CHR research studies and clinical programs worldwide. The scales are prominently featured in large multi-site studies (eg, NAPLS-2, CAPR, AMP-SCZ, MAPS), single-site projects, and in numerous systematic reviews and meta-analyses, highlighting their widespread adoption. The original Cornblatt et al.’s^7^ paper has garnered over 740 citations per Google Scholar, although citation count alone cannot capture the scales’ true clinical and research impact.

This measure represents just one of Dr. Cornblatt and her team’s many influential contributions to psychosis research. Her recent passing offers an opportunity to reflect on her legacy and the lasting impact of the tools she helped pioneer. Our goal is to provide a review of the GF scales’ legacy in psychosis risk and early psychosis intervention research, attempting to document and honor the scales’ impact on psychosocial treatment in the field. An AI-based tool (Elicit) was used to conduct data extraction from the peer-reviewed literature citing Cornblatt et al.^7^ The aims of this review include:

(1) To quantify and describe the scale’s usage (total citations, citations with explicit scale usage, scale usage in psychosocial interventions specifically).
(2) To summarize key findings from studies employing the scales within the context of psychosocial intervention, highlighting their contribution to our understanding of social and role functioning outcomes in CHR and early psychosis populations.

Collectively, this work will offer a synthesis of the GF scales, providing a picture of their reach and influence in the literature.

## Methods

Citation data for articles referencing the GF: Social and GF: Role scales^7^ were collected via Google Scholar in July 2025. We selected Google Scholar because it indexes a broad range of disciplines and publication types and therefore provides wide citation coverage for the Cornblatt et al. article; we then restricted our sample to peer-reviewed journal articles through manual screening and application of predefined inclusion criteria. To be eligible for inclusion, articles were required to meet the following criteria: (1) published in a peer-reviewed, English-language academic journal; (2) reported empirical results involving the use of either the GF: Social or GF: Role scale as a primary or secondary measure; and (3) provided full-text access at the time of review. Grey literature, including conference proceedings, dissertations, and preprints, was excluded. Additionally, articles primarily focused on theoretical discussion or commentary without empirical data were excluded.

**Figure 1:**
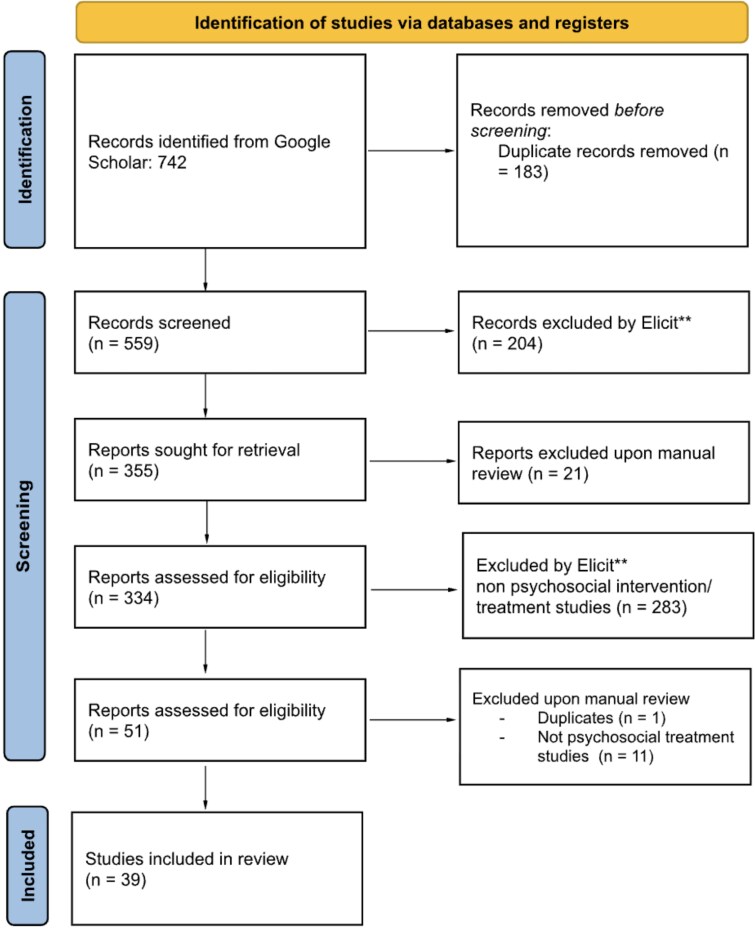
PRISMA Diagram.

The initial title and abstract screening were performed manually by the research team in July 2025. Following title and abstract screening, a full-text review was conducted. In recognition of emerging research and guidance on the use of artificial intelligence (AI) tools to support reviews,^23^^,^^24^ this review incorporated the use of Elicit, an AI-powered tool designed to assist with literature reviews, during the data extraction portion of the review. Past research has demonstrated that Elicit, and similar AI-based software, can greatly improve the speed of the review process.^25^^,^^26^ In alignment with past recommendations, this review employed manual oversight by the research team during the extraction process.^26^ Specifically, Elicit was used to conduct preliminary data extraction for included articles. All AI-generated outputs were then reviewed for accuracy and completeness by human coders in August 2025. Corrections were made as necessary based on manual verification of the full-text articles. A PRISMA flow diagram^27^ illustrating the screening process is presented in Figure 1.

For each study, we extracted article information (i.e., title, authors, journal, abstract), country of origin, sample characteristics, study objectives, study design, study setting(s), implementation of the GF: Social and GF: Role scales (i.e., purpose, scoring, administration information), measures used, analytic approach, social and role functioning outcomes, key findings, limitations, and funding sources using Elicit. All data were entered into a structured spreadsheet using a standardized codebook. Human coders then reviewed extracted article information for accuracy; corrections to extracted information were made where necessary to the final sample of 39 psychosocial treatment studies that used the GF: Social and GF: Role scales.

## Results

As outlined in the PRISMA Diagram in Figure 1, a total of 742 records were identified via Google Scholar as having cited Cornblatt and colleagues^7^ original article introducing the GF: Social and GF: Role scales. Duplicate records (*n* = 183) were removed via Elicit software, leaving 559 to be screened for use of the GF: Social and GF: Role scales. We then queried Elicit to identify articles that referenced Cornblatt et al.^7^ and explicitly used the GF: Social and/or GF: Role scales. Elicit identified 355 of 559 records as articles that used GF: Social and/or GF: Role scales (excluding 204 records that cited Cornblatt et al.^7^ but did not use the GF: Social and/or GF: Role scales), and then extracted article-specific data from these 355 citations (information extracted described above). Upon manual coding and review, 334 citations were confirmed to have used and reported quantitative data involving the GF: Social and/or GF: Role scales. We then queried Elicit to identify articles that specifically examined a psychosocial intervention. Of the 334 citations that used the GF: Social and/or GF: Role scales, Elicit identified 51 of these citations as psychosocial treatment or intervention studies. Manual coding and review of full texts yielded a final sample of 39 citations that used the GF: Social and/or GF: Role scales in a psychosocial intervention study. See Figure 1 for complete PRISMA Diagram.

**Figure 2:**
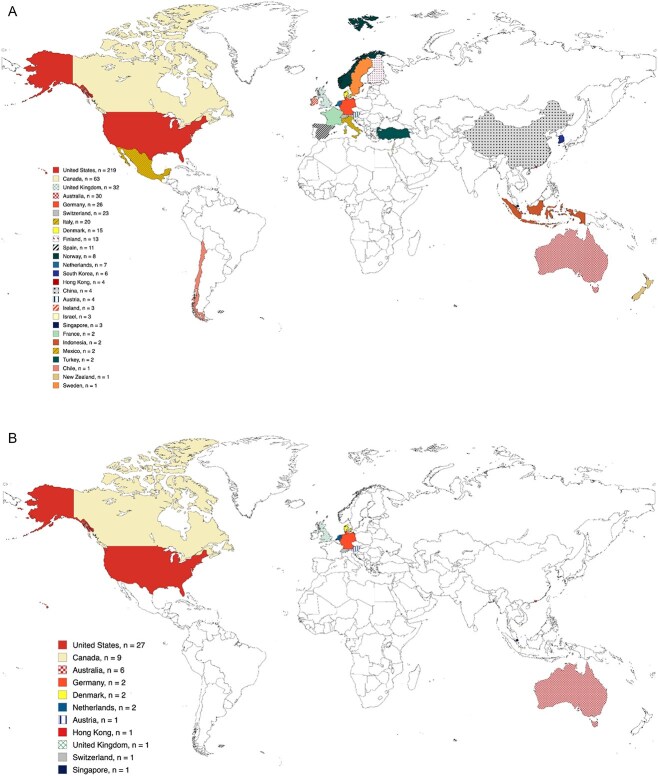
(A) Geographic Distribution of All Peer-Reviewed Published Articles (*n* = 334) Using the GF:S and GF:R. Due to the presence of multi-site studies, some studies may be represented multiple times. Six articles did not report the study location. (B) Geographic distribution of peer-reviewed published articles (*n* = 39) that used the GF:S and GF:R in treatment or intervention studies. Due to the presence of multi-site studies, some studies may be represented multiple times.

From the larger sample of 334 articles, the GF:S, GF:R, or both were used in studies conducted in 26 countries (see Figure 2). Among the included 39 psychosocial intervention studies, the GF:S, GF:R, or both were used in studies across 11 countries (see Figure 2).

Twenty-two of the included psychosocial intervention studies examined CHR samples specifically, 12 examined first episode psychosis (FEP) samples, four examined mixed samples (eg, CHR and FEP), and one included study focused on individuals with non-psychosis-spectrum symptoms.

Psychosocial interventions spanned multiple treatment delivery formats, including coordinated specialty care (CSC), individual therapy, family-based treatment, group therapy, individual coaching, vocational coaching, and computer-assisted intervention. Interventions employed within FEP, mixed, and non-psychosis-spectrum samples are presented in Tables 1-3. Interventions employed within CHR samples are presented in Table 4. Results of psychosocial treatment studies that used the GF:S and/or GF:R are discussed below.

### Clinical High-Risk

Outcomes across studies examining interventions in CHR populations were mixed, with modest evidence supporting improvements in social functioning and more limited gains in role functioning (Table 4). Several small to moderate-sized studies reported significant improvements on the Global Functioning: Social (GF: Social) scale, particularly in trials that employed psychosocial or group-based interventions. For example, CLUES,^28^ a 6-month cognitive remediation and social skills program involving 17 participants (82.4% male; *M*_age_ = 20.53), led to significant improvements in social functioning. Similarly, MOMENTUM,^29^ an online strengths- and mindfulness-based social therapy delivered to a sample of 14 individuals (78% female; *M*_age_ = 20.3) with a 2-month follow-up period, demonstrated positive gains in social functioning. Landa et al.^30^ reported improved social functioning following 15 weeks of group-and-family-based CBT (GF-CBT) in a small sample (*n* = 6; 66.7% female; *M*_age_ = 19.5). In a larger randomized controlled trial, Addington et al.^31^ evaluated the effects of cognitive-behavioral social skills training (CBSST) across 152 youth (*M*_age_ = 17.4), finding significant within-group improvements on the GF: Social scale at a 12-month follow-up, although no between-group differences emerged when compared to a supportive therapy condition. Devoe et al.,^32^ reported that participants receiving either CBSST or supportive therapy showed that improvements in social functioning were mediated by reductions in attenuated psychotic symptoms, rather than being directly attributable to the intervention. A pilot study by Malda et al.^33^ targeting supported education and employment in youth at CHR reported significant improvements in both GF: Role and GF: Social. By contrast, numerous interventions produced no statistically significant improvements in social functioning. These included BEGIN, a 5-week psychoeducational program for CHR individuals (*n* = 26; *M*_age_ = 20.42),^34^ and a feasibility trial combining transcranial direct current stimulation with CBT over 3 weeks (*n* = 5).^35^ Similarly, a 4-week biofeedback-based intervention with CBT elements, followed by a 4-week follow-up, showed no improvements in a sample of 20 youth.^36^ Larger multisite trials also yielded null findings. In the NEURAPRO trial,^37^ 304 participants (*M*_age_ = 19.1) were randomized to receive either omega-3 polyunsaturated fatty acids or placebo alongside case management over a 6-month intervention and follow-up period; no group differences in social functioning were found. Similarly, the FOCUS trial,^38^ which included 146 participants over a 20-week cognitive remediation intervention with 6- and 12-month follow-ups, found no significant improvements. In another large-scale RCT (ReGroup),^39^ 203 youth (*M*_age_ = 17.4) received an 18-week group-based intervention.

**Table 1:** Psychosocial Intervention Studies using the GF: Social and GF: Role Scales in FEP Samples (*n* = 12)

**Article title**	**Authors**	**Year**	**Sample information** ^a^	**Intervention name**	**Length of intervention/follow-ups**	**GF: social scale results**	**GF: role scale results**
Neuroplasticity-based auditory training via laptop computer improves cognition in young individuals with recent onset schizophrenia.	Fisher et al.^40^	2015	*n* = 86, AT *n* = 43, CG *n* = 43 **AT:** *M*_age_ = 21.70 (SD = 3.26); 72.1% Male, 27.9% Female **CG:** *M*_age_ = 20.74 (SD = 3.37); 76.7% male, 23.3% female	Cognitive training	8 weeks of training; Post-training assessments	No significant change	No significant change
The impact of premorbid adjustment, neurocognition, and depression on social and role functioning in patients in an early psychosis treatment program	Minor et al.^41^	2015	*N* = 47 (*n* = 47 with GF:R at both time points, *n* = 46 with GF:S at both time points); *M*_age_ 22 (SD = 3.5); 70.2% Male; 47.8% non-Hispanic Black, 34.8% non-Hispanic White, 6.3% East or Southeast Asian, 8.7% Multiracial, 2.2% Other	PREP^R^ early psychosis specialty services	6 months; baseline and 6-month follow-up assessments	Significant improvement	Significant improvement
A randomized, controlled trial of Social Cognition and Interaction Training (SCIT) for outpatients with schizophrenia spectrum disorders	Roberts et al.^42^	2014	*n* = 66, SCIT *n* = 33, TAU *n* = 33; **SCIT:** *M*_age_ = 40 (SD = 12.2); 66.7% Male, 33.3% Female; 54.6% Caucasian, 45.5% African American, 3% Hispanic/Latino **TAU:** *M*_age_ = 39.4 (SD = 10.8); 66.7% male, 33.3% female; 72.7% White, 27.3% African-American, 9.7% Hispanic/Latino	SCIT	20-24 weeks; post-treatment, 3-month follow-up	Significant improvement at post-treatment and follow-up for SCIT compared to TAU	N/A
A randomized controlled trial of cognitive remediation and long-acting injectable risperidone after a first episode of schizophrenia: improving cognition and work/school functioning	Nuechterlein et al.^43^	2022	*N* = 60, CR/RLAI *n* = 12, CR/Oral Ris *n* = 17, HBT/RLAI *n* = 17, HBT/Oral Ris *n* = 14; **CR/RLAI:** *M*_age_ = 21.7; 83% male; 50% Caucasian, 25% African-American, 25% Asian, 33% Hispanic; **CR/Oral Ris:** *M*_age_ = 21.4, 71% male; 59% Caucasian, 29% African-American, 6% Asian, 6% Native American, 53% Hispanic **HBT/RLAI:** *M*_age_ = 22.8; 82% male; 47% Caucasian, 41% African-American, 12% Asian, 35% Hispanic **HBT/Oral Ris:** *M*_age_ = 20.9, 71% male; 71% Caucasian, 14% African-American, 7% Asian, 7% Mixed, 43% Hispanic	Cognitive remediation therapy + long-acting injectable Risperidone	12 months; assessments at baseline, 6 months, and 12 months (or end of intervention)	N/A	Significant improvement in RLAIcompared to Oral risperidone; significantly larger improvement with CR compared to HBT
Personalizing interventions using real-world interactions: Improving symptoms and social functioning in schizophrenia with tailored metacognitive therapy	Minor et al.^44^	2022	*N* = 20; Standard MERIT *n* = 8, Tailored MERIT *n* = 12 **Standard MERIT:** *M*_age_ = 45.88 (SD =8.81); 50% Male, 50% Female; 75% African American, 25% Caucasian **Tailored MERIT:** *M*_age_ = 43.17 (SD = 12.43); 41.7% male, 58.3% female; 75% African American, 25% Caucasian	MERIT captured by EAR	6 months (24 sessions), post-intervention follow-up	No significant change	N/A
Durable cognitive gains and symptom improvement are observed in individuals with recent-onset schizophrenia 6 months after a randomized trial of auditory training completed remotely	Loewy et al.^45^	2022	*n* = 145, AT *n* = 80, CG *n* = 65; **AT:** *M*_age_ = 21.69 (SD = 4); 76.2% male, 23.8% female **CG:** *M*_age_ = 20.51 (SD = 3.48); 72.3% male, 27.7% female	Cognitive training	8-weeks; assessment post-training +6- month follow-up (~8 months total from baseline)	No significant change	No significant change
Social cognition training as an intervention for improving functional outcome in first-episode psychosis: a feasibility study	Bartholomeusz et al.^46^	2013	*N* = 12; *M*_age_ = 21.6 (SD = 2.9); 41.7% male, 58.3% female	SCIT	10 weeks; Assessment Post-intervention	No significant change	Significant improvement
Family aided community treatment for the treatment of early psychosis: a proof of concept study	Melton et al.^47^	2016	*N* = 8; *M*_age_ = 19.6 (SD = 3.28); 75% male; 75% White, 25% Hispanic	FACT	12 months; 12 month follow-up	Significant improvement	No significant change
A novel, online social cognitive training program for young adults with schizophrenia: A pilot study	Nahum et al.^48^	2014	*N* = 34, SZ *n* = 17, HC *n* = 17; **SZ:** *M*_age_ = 23.8 (SD = 3.2); 76.5% male, 23.5% female **HC:** *M*_age_ = 23.6 (SD = 3.6); 70.6% Male, 29.4% Female	SocialVille	6-12 weeks; post-training follow-up assessment	Significant improvement	No significant change
Aerobic exercise enhances cognitive training effects in first-episode schizophrenia: randomized clinical trial demonstrates cognitive and functional gains	Nuechterlein et al.^49^	2023	*N* = 47, CT&E *n* = 24, CT alone *n* = 23 **CT&E:** *M*_age_ = 22 (SD = 3.3); 54% male; 29% Caucasian, 25% African American, 4% Asian, 42% Mixed, 46% Hispanic **CT alone:** *M*_age_ = 22.8 (SD = 4.6); 87% Male; 22% Caucasian, 30% African American, 4% Native American, 44% Mixed, 35% Hispanic	Cognitive training and aerobic exercise	6 months; baseline, 3-months, 6-months (end of treatment period)	No significant change	Significant improvement in CT&E compared to CT alone at 6 months
Longitudinal determinants of client treatment satisfaction in an intensive first-episode psychosis treatment programme	Cruz et al.^50^	2017	*n* = 44, *M*_age_ = 22.02 (SD = 3.33), 66% male, 34% female, 7% Hispanic/Latino,5% Asian, 44% Black/African American, 34% White, 12% Multiracial, 5% “Other”	Prevention and Recovery in Early Psychosis Program (PREP)	6 months; baseline and post-intervention	Significant improvement	Significant improvement
A multivariate neuromonitoring approach to neuroplasticity-based computerized cognitive training in recent onset psychosis	Haas et al.^51^	2021	**PRONIA study diagnostic sample:** *n* = 91; *n* = 35, ROP *n* = HC *n* = 56; ROP *M*_age_ = 30.43; HC *M*_age_ = 30.64 **CCT intervention sample:** *n* = 26; Maintainers EMT *n* = 14, Improvers EMT *n* = 12; Maintainers EMT *M*_age_ = 27.46, Improvers EMT *M*_age_ = 26.10	CCT	5 weeks; baseline and post-intervention	Baseline only	Baseline only

Abbreviations: AT = auditory training; CCT = computerized cognitive training; CG = computer games; CR = cognitive remediation; CT&E = cognitive training and exercise; CT = cognitive training; EAR = Electronically Activated Recorder; EMT = Emotion Matching Task; FACT = family aided community treatment; HBT = Healthy Behaviors Training; HC = healthy control; MERIT = Metacognitive Reflection and Insight Therapy; Oral Ris = oral risperidone; PREP^R^ = Prevention and Recovery in Early Psychosis program; RLAI = long-acting injectable risperidone; ROP = recent onset psychosis; SCIT = Social Cognition and Interaction Training; SZ = schizophrenia.

^a^Where sociodemographic data is not included in the table, it was not reported in the study.

**Table 2:** Psychosocial Intervention Studies using the GF: Social and GF: Role Scales in Mixed Psychosis-Spectrum Samples (*n* = 4)

**Article title**	**Authors**	**Year**	**Sample information** ^a^	**Intervention name**	**Length of intervention/follow-ups**	**GF: social scale results**	**GF: role scale results**
Treatment outcomes for young people at clinical high risk for psychosis: data from a specialized clinic	West et al.^52^	2022b	*n* = 123; *M*_age_ = 20.1; 60.2% Cis-male, 32.5% Cis-female, 0.8% Trans-male, 2.4% Trans-female, 4.1% Other; 53.7% White, 14.6% Other/Mixed; 13.0% Black, 9.8% Asian, 7.3% Latinx, 1.6% Missing	Stepped care within CSC	Up to 2 years	Significant improvement	No significant change
Family-focused treatment for adolescents and young adults at high risk for psychosis: results of a randomized trial	Miklowitz et al.^53^	2014	*n* = 129, EC *n* = 63, FFT-CHR *n* = 66 **EC:** *M*_age_ = 17.4 (SD = 3.9); 44.4% Female; 23.8% Hispanic, 69.8% White, 6.3% Asian American, 15.9% African American, 1.6% Native American, 4.8% Other; **FFT-CHR:** *M*_age_ = 17.3 (SD = 4.2); 40.1% Female; 19.7% Hispanic, 68.2% White, 4.5% Asian American, 15.2% African American, 3% Native American, 7.6% Other	FFT-CHR	6 months; baseline and 6-month follow-up	No treatment group differences in improvement in social functioning	Greater improvements in role functioning in young adults (age ≧20 years) who received FFT-CHR compared to EC; improvements in role functioning in adolescents (age 16-19 years) greater in EC than in FFT-CHR; no treatment group difference among younger adolescents (12-15 years)
Clinical and functional outcomes after 2 years in the early detection and intervention for the prevention of psychosis multisite effectiveness trial	McFarlane et al.^54^	2015	*n* = 337, CLR *n* = 87, CHR *n* = 205, EFEP *n* = 45; **CLR:** *M*_age_ = 16.23 (SD = 3.18); 30% female; 71% Caucasian, 6% African-American, 5% Asian-American, 9% Hispanic **CHR:** *M*_age_ = 16.40 (SD = 3.30); 43% female; 61% Caucasian, 8% African-American, 4% Asian-American,17 % Hispanic; **EFEP:** *M*_age_ = 17.93 (SD = 3.10); 42% female; 47% Caucasian, 22% African-American, 0% Asian-American, 16% Hispanic;	EDIPPP (FACT model)	Assessments at 6-months, 12-months, 24-months	Significant improvement for CHR and EFEP groups compared to CLR	Significant improvement for CHR and EFEP groups compared to CLR
Uncharted waters: treating trauma symptoms in the context of early psychosis	Folk et al.^55^	2019	*N* = 22, Stage 1 *n* = 12, Stage 2 *n* = 10 **Stage 1:** *M*_age_ = 19.1 (SD = 5.4); 58% female, 42% Male; 50% Black, 17% White, 8% Pacific Islander, 25% Other Race, 33% Hispanic/Latino **Stage 2:** *M*_age_ = 16.8 (SD = 4.9); 70% female, 30% male; 40% Black, 20% White,10% Asian, 30% Other Race, 30% Hispanic/Latino	TI-CBTp	Assessments at baseline, 6-months, 12-months	No significant change	No significant change

Abbreviations: CHR = clinically higher risk; CLR = clinically lower risk; CSC = coordinated specialty care; EC = Enhanced Care; EDIPPP = Early Detection and Intervention for the Prevention of Psychosis Program; EFEP = early first-episode psychosis; FACT = Family-aided Assertive Community Treatment; FFT-CHR = family-focused therapy for individuals at clinical high risk; TI-CBTp = Trauma-Integrated Cognitive Behavioral Therapy for Psychosis.

^a^Where sociodemographic data is not included in the table, it was not reported in the study.

In contrast to social functioning, role functioning, as measured by the Global Functioning: Role (GF: Role) scale, showed fewer and less consistent gains. The CBSST intervention^31^ resulted in significant within-group improvements role functioning at 12 months, though, again, no between-group differences were found. Most other interventions did not yield significant changes in role outcomes. For instance, despite observed improvements in social functioning, the CLUES study^28^ found no corresponding gains in role functioning. Similarly, the InVEST trial,^56^ involving individualized vocational and educational support for 135 participants (*M*_age_ = 19.88), reported no significant group differences in role outcomes after a 6-month follow-up. FFT-CHR,^57^ a family-focused therapy trial involving 128 participants (*M*_age_ ~ 17.3), also did not find a statistically significant effect of treatment on role functioning, though the study aim was to examine moderating effects of sociodemographic factors on treatment outcomes.

Where improvements in role functioning were observed, they were typically limited in scope or failed to replicate across conditions or follow-up points. For instance, improvements were limited in the study by Piskulic et al.,^58^ where 32 CHR participants were randomized to cognitive remediation (Brain Fitness) or a computer game condition over a 10 to 12-week period.

### First Episode Psychosis

Outcomes from studies of individuals with FEP revealed mixed evidence of functional improvement (Table 1). Significant gains in social functioning were observed in studies employing either psychosocial or family-based interventions. Minor et al.^41^ examined a 6-month early psychosis specialty service program in a sample of 47 young adults (70.2% male; age 16-30, *M*_age_ = 22) and found improvements in both social and role domains. Roberts et al.^42^ conducted a randomized trial of Social Cognition and Interaction Training (SCIT) in a sample of 66 participants (*M*_age_ = 40), finding significant improvements in GF: Social scores at both post-treatment and 3-month follow-up. Melton et al.^47^ assessed Family Aided Community Treatment in eight FEP participants (*M*_age_ = 19.6), also reporting gains in social but not role functioning. Minor et al.^44^ evaluating a metacognitive therapy in a sample of 20 adults (*M*_age_ = 44.25), found no changes in social functioning.

A study by Nahum and colleagues^48^ evaluated SocialVille, a neuroplasticity-based online social cognitive training program, in 34 young adults (17 with schizophrenia and 17 healthy controls; schizophrenia group *M*_age_ = 23.8, 76.5% male) and found significant improvements in social functioning at post-training and follow-up. Several trials involving neuroplasticity-based cognitive training failed to yield improvements in social functioning. These include a study by Fisher et al.^40^ involving 86 recent-onset patients with schizophrenia (*M*_age_ ~ 21; 74.4% male), and a 6-month follow-up trial by Loewy et al.^59^ with 145 participants, both of which showed no significant post-intervention change.

Role functioning, by contrast, appeared more responsive to treatment. Nuechterlein et al.^43^ reported significant improvements in role outcomes following a 12-month combined intervention of cognitive remediation and long-acting risperidone in a sample of 60 participants (*M*_age_ = 21.7; 77% male). A more recent study by Nuechterlein et al.^49^ found that aerobic exercise enhanced the effects of cognitive training, resulting in greater improvements in role functioning after 6 months in a sample of 47 participants (*M*_age_ = 22.4; 70% male). Nevertheless, other studies, such as SCIT^42^ and SocialVille^48^ (*n* = 34), either did not assess role functioning or found no significant changes.

Overall, interventions for FEP populations were more likely to produce improvements in role functioning when pharmacological and psychosocial approaches were integrated. Still, findings varied, and several interventions did not yield functional gains, underscoring the need for more targeted and sustained approaches.

**Table 3:** Psychosocial Intervention Studies using the GF: Social and GF: Role Scales in Non-Psychosis Samples (*n* = 1)

**Article title**	**Authors**	**Year**	**Sample information** ^a^	**Intervention name**	**Length of intervention/follow-ups**	**GF: social scale results**	**GF: role scale results**
Social cognitive training in adolescents with chromosome 22q11.2 deletion syndrome: feasibility and preliminary effects of the intervention	Shashi et al.^60^	2015	*N* = 22, intervention *n* = 13, control *n* = 9 **Intervention:** *M*_age_ = 14.84 (SD = 1.44); 50% female; 83% Caucasian **Control:** *M*_age_ = 14.02 (SD = 2.12); 57% Female; 100% Caucasian	SCT	26-weeks; Pre-intervention assessment, post-intervention assessment	No significant change	N/A

Abbreviation: SCT = Social Cognitive Training.

^a^Where sociodemographic data is not included in the table, it was not reported in the study.

**Table 4:** Psychosocial Intervention Studies using the GF: Social and GF: Role Scales in CHR Samples (*n* = 22).

**Article title**	**Authors**	**Year**	**Sample information** ^**a**^	**Intervention name**	**Length of intervention/follow-ups**	**GF: social scale results**	**GF: role scale results**
A pilot study investigating the effect of the BEGIN psychoeducation intervention for people at clinical high risk for psychosis on emotional and stigma-related experiences	Mikelic et al.^34^	2024	*N* = 26; *M*_age_ = 20.42 (12-35); 57.7% Female; 30.77% White, 38.46% Black, 19.23% Asian, 11.54% Other, 19.23% Mixed race	BEGIN	5 weeks	Baseline only	Baseline only
Treatment choices for youth at clinical high risk for psychosis: outcomes of a naturalistic study	Addington et al.^61^	2025	*n* = 50; *M*_age_ = 18.82 (SD = 4.48); 58% female, 42% male; 14% indigenous, 2% East Asian, 8% South Asian, 6% Black, 58% White, 12% Interracial	Psychoeducation + CBT + Medication	2-6 month treatment; 2, 6, 12, and 18 months follow-up	Baseline only	Baseline only
Cognitive behavioural social skills training: methods of a randomized controlled trial for youth at risk of psychosis	Addington et al.^39^	2021	*n* = 203; *M*_age_ = 17.4 (12-30); 51.2% female, 48.8% male; 62.1% Caucasian, 13.8% Asian, 10.3% Black, 13.8% Other	ReGroup	18 weeks; 12-month follow-up	Baseline only	Baseline only
White blood cell proportions are associated with response to psychosocial therapy in young people at ultra-high risk for psychosis	Barker et al.^62^	2025	*n* = 91, Remitters *n* = 31, Nonremitters *n* = 60; **Remitters:** *M*_age_ = 17.4 (SD = 3.6); 58.1% Male, 41.9% Female; 83.9% Genetically Inferred European Ancestry **Nonremitters:** *n* = 60; *M*_age_ = 16.8 (SD = 2.8); 58.3% Female; 75.0% Genetically Inferred European Ancestry	STEP clinical trial, psychosocial therapy	6 - 26 weeks; 4, 6, and 24 weeks	Baseline only	Baseline only
An integrated neurocognitive and social-cognitive treatment for youth at clinical high risk for psychosis: cognition for learning and for understanding everyday social situations (CLUES)	Friedman-Yakoobian et al.^28^	2019	*n* = 17; *M*_age_ = 20.53 (SD = 4); 82.4% male, 17.6% female; 70.6% White, 17.6% Black or African American, 11.8% Interracial	CLUES	6 months	Significant improvement	No significant change
tDCS for the treatment of negative symptoms in youth at clinical-high-risk for psychosis: a feasibility study	Metzak et al.^35^	2024	*N* = 5; 60% female, 40% male; *M*_age_ = 18; 60% White, 20% 1st Nation, 20% mixed race	tDCS + CBT	3 weeks	No significant change	No significant change
Biofeedback to treat anxiety in young people at clinical high risk for developing psychosis	McAusland et al.^36^	2018	*N* = 20; *M*_age_ = 16.7; 70% female, 30% male	HRV biofeedback + CBT elements	4 weeks; 4 week follow-up	No significant change	No significant change
Enhancing social functioning in young people at Ultra High Risk (UHR) for psychosis: A pilot study of a novel strengths and mindfulness-based online social therapy	Alvarez-Jimenez et al.^29^	2018	*n* = 14; *M*_age_ = 20.3 (SD = 3.4); 78% female	MOMENTUM	2-month follow-up	Significant improvement	N/A
Neurocognitive and social cognitive training for youth at clinical high risk (CHR) for psychosis: a randomized controlled feasibility trial supportive therapy in youth at clinical high risk for psychosis	Friedman-Yakoobian et al.^63^	2022	*n* = 38, CLUES *n* = 20, EnACT *n* = 18; **CLUES:** *M*_age_ = 19.2; 55% male, 30% female, 15% Other; 60% White, 10% African American, 20% Interracial, 5% Asian, 5% did not disclose **EnACT:** *M*_age_ = 19.1; 66.7%% male, 22.2% female, 11.1% Other; 50% White, 22.2% African American, 11.1% Interracial, ~16.7% Asian	CLUES	~6 weeks; 3-month follow-up	CLUES showed significant improvements compared to EnACT	No significant differences between CLUES and EnACT
Development of a group and family-based cognitive behavioural therapy program for youth at risk for psychosis	Landa et al.^30^	2016	*N* = 6; *M*_age_ = 19.5; 66.7% female; 50% Black, 16.7% Hispanic/Latino, 16.7% White, 16.7% Mixed	GF-CBT for youth at risk for psychosis	15 weeks +3-month follow-up	Significant improvement	No significant improvement
Individualized vocational and educational support and training for youth at clinical high risk for psychosis	West et al.^56^	2022a	*n* = 135, InVEST *n* = 47, Non-InVEST *n* = 88 **InVEST:** *M*_age_ = 20.5; 63.8% Male; 55.6% White, 13.3% Asian, 11.1% Black, 10.2% Mixed, 3.9% other **Non-InVEST:** *M*_age_ = 19.88; 60.2% male; 56.6% White, 15.7% Black, 9.6% Hispanic/Latino, 7.2% Asian, 7.2% Mixed, 3.6% other	InVEST	6-month follow-up	No significant differences across groups	No significant differences across groups
Race/ethnicity and socioeconomic status as predictors of outcome following family therapy in youth at clinical high risk for psychosis	Ruiz-Yu et al.^57^	2024	*n* = 128, POC *n* = 64, NHW *n* = 64; **POC:** *M*_age_ = 17.63 (SD = 4.27); 59.4% female; 35.9% Hispanic/Latinx, 28.1% African American, 17.2% Multiracial, 10.9% Asian, 4.7% Native American, 1.6% Middle Eastern, 1.6% Other **NHW:** *M*_age_ = 17.05 (SD =3.84); 54.7% female	FFT-CHR	6 months; 6-month follow-up	No effect of race/ethnicity on change	No effect of race/ethnicity on change
A sequential adaptive intervention strategy targeting remission and functional recovery in young people at ultrahigh risk of psychosis	McGorry et al.^64^	2023	*n* = 342; *M*_age_ = 17.7 (SD = 3.1); 57.9% female, 42.1 % male	Stepped-care + case management	6 months	No significant difference in mean scores at 6 months between intervention groups	No significant difference in mean scores at 6 months between intervention groups
Pilot study of cognitive remediation therapy on cognition in young people at clinical high risk of psychosis	Piskulic et al.^58^	2015	*n* = 32, BF *n* = 18, CG *n* = 14 **BF:** *M*_age_ = 19.72 (SD = 5.71); 61.1% male, 38.9% female **CG:** *M*_age_ = 17.50 (SD = 3.48); 71.4% Male, 28.6% Female	CRT	10-12 weeks; 3 and 9-month follow ups (post-baseline)	Significant differences at 6 months for BF group; No significant differences at 6 months for CG group	No significant group differences at 6 months for either BF group or CG group
Cognitive-behavioral social skills training: outcome of a randomized controlled trial for youth at risk of psychosis	Addington et al.^31^	2023	*n* = 152, CBSST *n* = 70; ST *n* = 82; **CBSST:** *M*_age_ = 17.36; 41.4% male, 58.6% female; 58.6% Caucasian, 11.4% Black, 30% Other **ST:** *M*_age_ = 17.49; 48.8% male, 51.2% female; 62.2% Caucasian, 11% Black, 26.8% Other	CBSST	18 weeks; 12-month follow-up	Significant improvement at 12- month follow-up for CBSST, not ST group; No significant difference in mean scores at 12 months between intervention groups	Significant improvement at 12- month follow-up for CBSST and ST groups; No significant difference in mean scores at 12 months between intervention groups
Cognitive-behavioural social skills training: mediation of treatment outcomes in a randomized controlled trial for youth at risk of psychosis	Devoe et al.^32^	2025	*N* = 152, CBSST *n* = 70, ST *n* = 82; **CBSST:** *M*_age_ = 17.36 (SD = 4.01); 41.4% male, 58.6% female; 58.6% Caucasian, 11.4% Black, 30% Other **ST:** *M*_age_ = 17.49 (SD = 4.12); 48.8% male, 51.2% female; 62.2% Caucasian, 11% Black, 26.8% Other	CBSST	18 weeks; 12-month follow-up	APS mediated the treatment effects on the outcomes of social functioning	APS mediated the treatment effects on the outcomes of role functioning
Effect of ω-3 polyunsaturated fatty acids in young people at ultrahigh risk for psychotic disorders: the NEURAPRO randomized clinical trial	McGorry et al.^37^	2017	*n* = 304, PUFA *n* = 153, Placebo *n* = 151; **PUFA:** *M*_age_ = 19.4 (SD = 4.8); 49% female; 80.4% Caucasian, 2% Black, 13.1% Asian, 3.3% Other, 1.3% Missing **Placebo:** *M*_age_ = 18.9 (SD = 4.3); 59.6% female; 80.1% Caucasian, 2.6% Black, 10.6% Asian, 3.3% Other, 3.3% Missing	CBCM + PUFA	6 months; 6 month follow-up	No significant difference in change at 6 months between groups; No significant difference in change at 6-month follow-up between groups	Greater change in placebo group compared to PUFA group at 6 months; No significant difference in change at 6-month follow-up between groups
Cognitive remediation plus standard treatment versus standard treatment alone for individuals at ultra-high risk of developing psychosis: Results of the FOCUS randomised clinical trial	Glenthøj et al.^38^	2020	*n* = 146, CRT *n* = 73, TAU *n* = 73; **CRT:** *M*_age_ = 23.93 (SD = 4.67); 52.1% female **TAU:** *M*_age_ = 23.90 (SD = 3.79); 60.3% female	CRT	40 h/20 weeks; 6-month (cessation of treatment) and 12-month follow-up	No significant change at 6- or 12-month follow-up in either CR + TAU or TAU group	No significant change at 6- or 12-month follow-up in either CR + TAU or TAU group
A pilot study of cognitive training in clinical high risk for psychosis: initial evidence of cognitive benefit	Hooker et al.^65^	2014	*n* = 28, CHR *n* = 14, HC *n* = 14; **CHR:** *M*_age_ = 21.9 (SD = 4.2); 50% female, 50% male **HC:** *M*_age_ = 24.1 (SD = 3.2); 57.1% Female, 42.9% Male	TCT	40 h/8 weeks; pre/post TCT and 1 month follow-up	No significant changes at end of intervention in either CHR or HC group	No significant changes at end of intervention in either CHR or HC group; greater improvement in processing speed pre-post TCT associated with greater improvement in role functioning
Intensive auditory cognitive training improves verbal memory in adolescents and young adults at clinical high risk for psychosis	Loewy et al.^59^	2016	*n* = 83, AT *n* = 50, CG *n* = 33; **AT:** *M*_age_ = 17.76 (SD = 3.06); 52% male, 48% female **CG:** *M*_age_ = 18.73 (SD = 4.60); 49% male, 51% female	Cognitive training	40 hours/8 weeks; 8 weeks (post-baseline)	No significant changes at end of intervention in either AT or CG group	No significant changes at end of intervention in either AT or CG group
Supported education and supported employment for individuals at clinical-high risk of psychosis: a pilot study	Malda et al.^33^	2023	*N* = 20, SEE *n* = 9, TAU *n* = 11; *M*_age_ = 21.7 (SD = 6.3); 95% female; 100% White	SEE	Baseline assessment, 12 month follow up	Greater improvement in social functioning after SEE intervention compared to TAU (moderate effect)	Greater improvement in role functioning after SEE intervention compared to TAU (moderate effect)
Enhancing Role functioning in clinical high risk for psychosis: an open trial of "InVEST" (individualised vocational and educational support and training)	West et al.^66^	2025	*N* = 5; *M*_age_ = 15; 20% cis-male, 20% cis-female, 20% agender, 20% preferred not to disclose gender; 20% Hispanic, 20% Mixed Race, 60% White	InVEST	16 weeks; four-month follow-up	N/A	Most participants showed improvement in role functioning

Abbreviations: AT = targeted auditory training program; BEGIN = Brief Educational Guide for Individuals in Need; BF = Brain Fitness; CBT = cognitive behavioral therapy; CBSST = cognitive-behavioral social skills training; CBCM = cognitive behavioral case management; CG = computer games; CLUES = Cognition for Learning and for Understanding Everyday Social Situations; CRT = Cognitive Remediation Therapy; EnACT = Enriched Acceptance and Commitment Therapy; FFT-CHR = family-focused therapy for CHRp youth; GF-CBT = group-and-family-based cognitive behavioural therapy; HRV Biofeedback = heart rate variability (HRV) biofeedback; InVEST = Individualized Vocational and Educational Support and Training; PUFA = polyunsaturated fatty acids; ReGroup = Recovery through Group; SEE = Supported Education and Supported Employment; STEP = Staged Treatment in Early Psychosis; TAU = treatment as usual; TCT = Targeted Cognitive Training; tDCS = transcranial direct current stimulation.

^a^Where sociodemographic data are not included in the table, it was not reported in the study.

### Mixed CHR/FEP

Four studies included mixed samples of CHR and FEP participants, and findings are summarized in Table 2. The most promising results emerged from interventions offering sustained, multi-component care. West et al.^52^ evaluated a stepped-care model within CSC in a sample of 123 individuals (*M*_age_ = 20.1) over a period of up to 2 years. Significant improvements were found in social functioning, although no corresponding gains were observed on the GF: Role scale. McFarlane et al.^54^ reported on outcomes from the Early Detection and Intervention for the Prevention of Psychosis Program, a large multisite study involving 337 youth (*M*_age_ = 16.6). Participants in the CHR and early FEP arms showed significant improvements in both social and role functioning at 6-, 12-, and 24-month follow-ups compared to the non-treatment low-risk group. However, group definitions varied across sites, limiting comparability. In contrast, Folk et al.^55^ evaluated trauma-integrated CBT for psychosis in a sample of 22 youth, including both FEP and CHR participants, and found no significant changes in social or role functioning at 6- and 12-month follow-ups. Mixed-sample studies (CHR and FEP) suggest that coordinated, multi-component care models may produce functional improvements in one or both domains, while smaller or single-focus interventions have yielded limited effects.

Across CHR samples, psychosocial interventions more often yielded gains in social functioning than in role functioning. In FEP, role functioning gains were at least as frequent, and sometimes more robust, particularly with integrated approaches (eg, cognitive remediation combined with pharmacological or exercise components).

## Discussion

The current synthesis represents a testament to the recognition of social and role functioning in the scientific literature. As Dr. Cornblatt, her colleagues, and countless others have recognized, the field of mental health research in general, and the subfield of working with people at CHR and across the early psychosis continuum, client functioning stands alongside clinical symptoms in terms of importance. Many might argue that functioning may even supersede symptomatology as a driver of quality of life and help seeking. The Global Functioning Social and Role Scales have provided the vehicle for researchers and clinicians to collect this information in a systematic, independent (social and role separately), and developmentally appropriate way. The qualities of the GF: Social and GF: Role scales dovetail and have potentiated mental health approaches that prioritize decision-making in conjunction with the client and client-driven care. From the perspective of the research community, the uptake of this measure has been high, with hundreds of articles identified as having cited the scales.

There are numerous ways the GF: Social and GF: Role scales have been used in the literature. To capture patterns across a large body of work, we used an AI-powered tool (Elicit) to assist with identifying and coding articles that cited and used the GF scales (Figure 1). In our review, we found that the reach of the Global Functioning Social and Role scales was vast, with the measure being used in 26 countries (Figure 2A) with hundreds of research participants and clients ranging in age from 12 to 60 years old. At the time of our search, the 334 included articles that used the GF: Social and/or GF: Role scales had been cited an average of 40.79 times. This highlights the visibility of this work in the broader literature. Along with the AI coding, these descriptive findings highlight the broad scope of the scales’ impact across prevention, intervention, and prediction research, suggesting a substantial impact of Dr. Cornblatt and her colleagues extending beyond the studies captured in this review.

Beyond just documenting the sheer scope of the GF scales, our review focused specifically on psychosocial treatment research. Across CHR and FEP populations, the scales were commonly used to assess psychosocial intervention outcomes, particularly at baseline and post-intervention time points. While the integration of these scales varied across studies, several protocols, such as CLUES,^28^^,^^63^ CBSST,^31^ and SCIT,^42^^,^^46^ effectively incorporated the GF scales and demonstrated treatment-related changes in functioning. Overall, findings suggested a preliminary domain- and stage-specific pattern of functional change. In CHR samples, gains were more apparent in social than in role functioning and were most common in trials that explicitly targeted social skills or social cognition. In FEP samples, role functioning appeared more responsive, especially in interventions that integrated psychosocial treatment with pharmacotherapy or structured exercise, whereas social functioning improved primarily when it was directly targeted, although these patterns should be interpreted cautiously given small sample sizes and heterogeneous designs. In contrast, role functioning showed fewer consistent gains across studies, with improvements typically limited to interventions that included targeted educational or vocational support or combined pharmacological and psychosocial components.

Some studies also indicated that functional improvements, particularly in social functioning, were mediated by reductions in clinical symptoms, such as attenuated positive symptoms,^32^ further indicating a complex relation between symptoms and functional outcomes. From a clinical standpoint, these findings support the utility of the GF scales in monitoring real-world, client-oriented treatment outcomes. They also highlight the value of integrating functional goals, such as school participation, work readiness, and social connectedness, into early intervention planning for youth at risk for psychosis or in the early stages of illness.

Social functioning is therefore a particularly compelling target for future intervention and assessment work in CHR and early psychosis. Adolescence and young adulthood, the developmental window that typically overlaps with the CHR period, is characterized by rapid expansion in social roles and expectations. Successfully navigating friendships, romantic relationships, family responsibilities, and early educational and occupational roles helps set the stage for adult life. Beyond treatment response, social functioning has repeatedly been linked to mechanisms thought to drive illness progression and to risk for transition to threshold psychosis, over and above related constructs such as social cognition, negative symptoms, and general cognitive functioning. As the field continues to move away from an exclusive emphasis on predicting transition and instead recognizes CHR as a treatment-seeking population in its own right, tracking and improving social and role outcomes becomes a central strategy for reducing long-term disability. There is also promise in extending this lens earlier, into premorbid periods when social skills are developing rapidly and show substantial variability.

The variability in outcomes observed across CHR studies highlights the need for more targeted and methodologically consistent research and need for more targeted interventions for functioning in this population. Unlike studies with individuals experiencing FEP, interventions in CHR samples often yielded mixed or limited improvements in functioning, particularly in role domains. This pattern may reflect several related factors, including heterogeneity in sample characteristics, variability in intervention intensity and duration, and the relatively early stage of illness in CHR populations. Compared with FEP cohorts, CHR samples in these trials typically enter with higher and less variable GF: Social and GF: Role scores, which leaves less room for measurable improvement and reduces power to detect change. In contrast, FEP interventions often enrolled individuals with more pronounced role impairment at baseline, so intensive multi-component treatments, particularly those that included pharmacotherapy, had greater opportunity to yield detectable gains in GF: Role and, in some cases, GF: Social.

Additionally, many CHR studies relied on relatively short follow-up periods in samples where only a minority are expected to progress to threshold psychosis or more severe symptomatology. In this context, modest or variable functional changes may in part reflect the heterogeneity of natural clinical trajectories, including youth whose symptoms improve or shift toward non-psychosis outcomes over time. Short follow-up periods, combined with the fact that many CHR studies were based on small sample sizes, limit the ability to detect lasting or generalizable effects. A more detailed characterization of functional trajectories in CHR, with attention to variable clinical progression and premorbid pathways, may clarify when and how early interventions can meaningfully alter long-term outcomes. In practice, this will require developmentally informed approaches that are explicitly anchored to the functional goals that at-risk youth and their families identify as most important.

Future work can take better advantage of rapidly evolving technologies to deepen understanding of social and role functioning in daily life. Smartphone-based ecological momentary assessment and passive sensing of mobility, communication patterns, and activity levels could be linked to GF: Social and GF: Role ratings to examine how moment-to-moment experiences accumulate into broader functional trajectories. Wearable devices and digital cognitive or skills-based training platforms may offer novel ways to track functional response between clinic visits and to tailor interventions to the specific social and role goals of youth at risk for or experiencing early psychosis.

### Limitations and Future Directions

Despite the wealth of studies employing the GF: Social and Role scales, more work can continue to strengthen the field’s understanding of functioning among people in early phases of psychosis and the psychometrics of the scales. Very few of the studies we coded contained enough information to draw any conclusions about cultural invariance and how the scales operate in different communities. Additionally, there is likely variability in administration and sites and studies, possibly impacting reliability. Notably, however, initial inter-rater reliability for GF:S and GF:R was excellent (ICCs generally ≥ 0.80), including cross-site ratings.^22^

The next generation of functional assessments can build upon Cornblatt et al.’s foundation by combining interviewer-rated scales with technology-enabled tools. For example, telehealth and online treatment platforms can embed brief, repeated assessments of social engagement and occupational or educational participation, while virtual or augmented reality environments can be used to practice and evaluate complex social and role demands in safe, graded ways. Digital tools can also support measurement-based care by feeding GF: Social and GF: Role scores, ecological momentary reports, and passive sensor data into decision-support systems that help clinicians and clients collaboratively set and monitor functional goals. Importantly, any expansion into digital assessment, whether or not it incorporates artificial intelligence, will require careful attention to validity, transparency, privacy, and equity so that new measures enhance, rather than undermine, the values that motivated the GF scales.

Greater attention to cultural responsiveness and fairness is especially critical to ensure that functional ratings do not conflate illness-related impairment with contextual disadvantage.^67^ New innovations within and beyond the GF scales may prove invaluable in a quickly evolving world. Normative patterns of social engagement and role participation in adolescents and young adults have changed quickly in the context of social media, online gaming, artificial intelligence tools, and remote work or online education. Measures developed for an earlier era may not fully capture where and how young people now form and maintain relationships or pursue educational and vocational goals. The wide variety of developmentally normative activities, combined with the characteristic heterogeneity of CHR presentations, further complicates efforts to summarize functioning in a single metric. Future iterations of the GF scales or related measures could maintain the efficiency of a global rating while also incorporating greater breadth and flexibility so that the varied tapestry of adolescent and young adult roles is better represented. Large-scale international efforts in early psychosis provide an opportunity to test and refine such tools across diverse settings, with a focus on predicting and improving functional outcomes rather than transition alone. Nonetheless, the GF: Social and Role scales remain among the most enduring legacies in CHR work, shaping intervention science and prediction models for psychosis. Furthermore, they accomplish many goals within this field using a client-centered and client-driven approach that aligns with lived experience and often the priorities of clients.

## Conclusion

The GF: Social and GF: Role scales have become essential tools for assessing functioning in youth and young adults at risk for or experiencing early psychosis. This review demonstrates their broad adoption across prevention, intervention, and prediction research, highlighting both their scientific and clinical impact. Findings across CHR and FEP studies using the GF: Social and GF: Role scales suggest that improvements in functioning are selective and context-dependent within this small and heterogeneous body of work. In CHR samples, social functioning showed modest gains, particularly in interventions that directly targeted social domains, whereas role functioning rarely changed. In FEP samples, there was somewhat more consistent evidence of role functioning improvement, especially in multi-component approaches that integrated psychosocial and pharmacological elements, with social functioning improving mainly when it was an explicit treatment target.

The widespread use of these measures underscores their enduring value as developmentally appropriate, client-centered assessments. Dr. Cornblatt and her colleagues’ vision to create developmentally anchored measures of social and role functioning has shaped research and clinical care in psychosis risk. Their contributions continue to guide intervention science, prediction models, and client-driven approaches that prioritize functional recovery alongside symptom reduction. The GF: Social and GF: Role scales remain a scientific legacy and a practical reminder of the importance of centering lived experience in mental health research and treatment.
